# Dietary Factors and Risk of Gout: A Two-Sample Mendelian Randomization Study

**DOI:** 10.3390/foods13081269

**Published:** 2024-04-21

**Authors:** Guosen Ou, Jialin Wu, Shiqi Wang, Yawen Jiang, Yaokang Chen, Jingwen Kong, Huachong Xu, Li Deng, Huan Zhao, Xiaoyin Chen, Lu Xu

**Affiliations:** School of Traditional Chinese Medicine, Jinan University, Guangzhou 510632, China; ouguosen@stu2022.jnu.edu.cn (G.O.); wujialin@stu2020.jnu.edu.cn (J.W.); sikeiwong@stu2018.jnu.edu.cn (S.W.); 5ahuman@stu2021.jnu.edu.cn (Y.J.); yiuhong@stu2022.jnu.edu.cn (Y.C.); vincentkjw@stu2022.jnu.edu.cn (J.K.); xuhuachong@jnu.edu.cn (H.X.); dengli@jnu.edu.cn (L.D.); zhaohuan@jnu.edu.cn (H.Z.); tchenxiaoyin@jnu.edu.cn (X.C.)

**Keywords:** gout, uric acid, mendelian randomization analysis, food, macronutrient

## Abstract

Background: Dietary intervention is the preferred approach for the prevention and clinical management of gout. Nevertheless, the existing evidence regarding the influence of specific foods on gout is insufficient. Methods: We used two-sample Mendelian randomization for genetic prediction to analyze the relationship between the intake of more than a dozen daily food items, such as pork, beef, cheese, and poultry, and dietary macronutrient intake (fat, protein, carbohydrates, and sugar) and the risk of developing gout and elevating the serum uric acid level. Inverse-variance weighted MR analyses were used as the main evaluation method, and the reliability of the results was tested by a sensitivity analysis. Results: Cheese intake was associated with lower serum uric acid levels, and tea intake (OR = 0.523, [95%CI: 0.348~0.784], *p* = 0.002), coffee intake (OR = 0.449, [95%CI: 0.229~0.882], *p* = 0.020), and dried fruit intake (OR = 0.533, [95%CI: 0.286~0.992], *p* = 0.047) showed a preventive effect on the risk of gouty attacks. In contrast, non-oily fish intake (β = 1.08, [95%CI: 0.24~1.92], *p* = 0.012) and sugar intake (β = 0.34, [95%CI: 0.03~0.64], *p* = 0.030) were risk factors for elevated serum uric acid levels, and alcohol intake frequency (OR = 1.422, [95%CI: 1.079~1.873], *p* = 0.012) was a risk factors for gout predisposition. Conclusions: These results will significantly contribute to the formulation and refinement of nutritional strategies tailored to patients afflicted with gout.

## 1. Introduction

Gout stands as the predominant cause of arthritis on a global scale, affecting over 41 million individuals worldwide [1]. Serum uric acid, identified as the primary risk factor for gout, and hyperuricemia exhibit prevalence rates exceeding 20% in developed nations like the United States and South Korea, and up to 16.4% in developing countries such as China [2]. While medications like colchicine, corticosteroids, non-steroidal anti-inflammatory drugs (NSAIDs), allopurinol, and febuxostat have traditionally been utilized for treating gout and hyperuricemia, these pharmaceutical interventions often accompany side effects, including but not limited to diarrhea, nausea, and potential liver and kidney complications [3]. Given the cautious approach warranted by the side effects associated with these medications in the management and prevention of gout, dietary interventions have garnered increased attention. This increased focus aims to lower uric acid levels and prevent gout by steering individuals away from high-purine foods, known sources that escalate uric acid production and stand as risk factors for these conditions [4]. However, the measurement of purine content is only one of the references in assessing whether a food is a risk factor or protective factor for gout and hyperuricemia. Certainly, the primary cause of elevated uric acid levels leading to hyperuricemia stems from impaired uric acid metabolism, particularly the reduced excretion of uric acid, rather than solely from the intake of exogenous purines [5]. It is notable that many determinations of food-related risk or protective factors are frequently derived exclusively from animal experiments and cross-sectional and retrospective studies. Relying solely on these methodologies may pose challenges in isolating and excluding other potential confounding factors that might interfere with experimental results [6]. Mendelian randomization (MR) analysis serves as a research methodology employed in deriving disease risk factors. It utilizes information derived from genome-wide association studies (GWAS) and leverages genetic variation to construct instrumental variables (IVs) for exposure. Through this method, the causal relationship between the exposure to certain factors and the subsequent disease outcomes can be estimated more accurately [7]. This methodology provides a more robust and potentially more reliable means to infer causation by using genetic variants as instrumental variables, providing insights into the effects of certain exposures on disease risk. Before conducting an MR analysis, it is imperative to meticulously screen the IVs to ensure that they meet three critical conditions: (1) The selected IVs must exhibit a robust correlation with the exposure factor under investigation, (2) IVs should only influence the outcome through the exposure factor being studied, ruling out any direct influence on the outcome independent of the exposure, and (3) IVs must not be correlated with confounding variables that could potentially distort the relationship between exposure and outcome [8]. Certainly, the use of single-nucleotide polymorphisms (SNPs) as analytical tools in an MR analysis is advantageous due to their independence from confounders and reverse causation. SNPs are randomly allocated at the time of conception, allowing for a more reliable basis for causal inference in research [8]. This advantage contributes to the heightened confidence in rigorously designed MR analyses, compensating for the scarcity of evidence arising from the challenges in conducting large-scale randomized controlled studies. Understanding the impact of dietary factors on serum uric acid level and gout in this way can help to develop dietary intervention strategies that contribute to the prevention and treatment of gout. Therefore, in this study, we used two-sample MR to analyze the causal effects of different food fractions on serum uric acid levels and gout. Going further, we also analyzed the impact of macronutrients (protein, sugar, carbohydrates, and fat) in foods on these diseases. Our findings will significantly assist in the development of tailored dietary strategies for individuals dealing with gout.

## 2. Methods

### 2.1. Exposure Phenotypic Data

**Figure 1:**
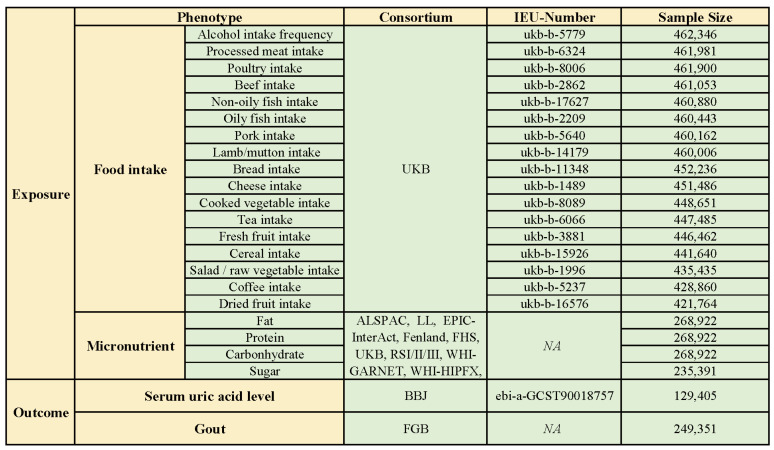
Exposure and outcome phenotypic data (ALSPAC: the Avon Longitudinal Study of Parents and Children; BBJ: Biobank Japan; DietGen: DietGen Consortium; EPIC: The European Prospective Investigation into Cancer and Nutrition study; FGB: FinnGen database; FHS: Framingham Heart Study; RSI/II/III: the Rotterdam Study I/II/III; UKB: UK Biobank; WHI-GARNET: Women’s Health Initiative—Genomics and Randomized Trials Network; WHI-HIPFX: Women’s Health Initiative—Hip Fracture GWAS; WHI-WHIMS+: Women’s Health Initiative—Memory Study).

Exposure phenotypic data related to food intake were obtained from Biobank UK (UKB), which is publicly available from the IEU Open GWAS program. These data are from a large prospective study involving genotypic and phenotypic data from over 500,000 individuals aged between 40 and 69 years in the UK [9]. In particular, food-intake-related GWAS data covered 17 common food items, including tea intake, coffee intake, beef intake, pork intake, lamb intake, poultry intake, processed meat intake, oily fish intake, non-oily fish intake, cheese intake, bread intake, salad/raw vegetable intake, cooked vegetable intake, dried fruit intake, alcohol intake frequency, cereal intake, and fresh fruit intake. SNPs significantly associated with the intake of macronutrients (protein, sugar, carbohydrate, and fat) from food were extracted directly from the literature of the GWAS study by Meddens SFW and colleagues. It is a meta-analysis of GWAS research encompassing European and American populations involving 12 cohorts with 268,922 participants (n_sugar_ = 235,391) (Figure 1) [10].

### 2.2. Outcome Phenotypic Data

To minimize bias in the study results due to sample overlap between outcome and exposure data, for gout-related GWAS data, we obtained data from the FinnGen database (R9). The FinnGen database integrates whole-genome sequencing data from an extensive cohort of more than 500,000 Finnish participants, of which the gout-related data cover 240,862 participants (n_case_ = 8489) [11]. Elevated serum uric acid level is a risk factor for gouty attacks; therefore, the effect of the above dietary factors on serum uric acid level was also evaluated in this study. Similarly, in order to avoid sample overlap, we chose GWAS data related to serum uric acid levels from Biobank Japan (BBJ) as one of the outcome indicators. During 2003–2008, BBJ collected DNA sequencing data, serum, and clinical information on 201,800 participants from 12 medical institutions in Japan. Of these, the sample size for data related to serum uric acid levels was 129,405, which was made publicly available through the IEU Open GWAS program (Figure 1).

### 2.3. Instrumental Variable Selection

Rigorous quality control measures have been implemented for the selection of IVs. Firstly, for the identification of SNPs significantly associated with the exposure phenotype, a threshold of *p* < 5 × 10^−8^ was employed as the selection criterion. Secondly, to eliminate linkage disequilibrium between SNPs, criteria of R^2^ < 0.001 and a window size >10,000 kb were utilized during the IV clumping procedure. Thirdly, SNPs with an effector allele frequency (EAF) < 0.01 were excluded from consideration. Fourthly, a coordination process for IVs was implemented to eliminate palindromic SNPs using the two-sample MR package. Fifth, in order to eliminate the influence of weak IVs on the analysis results, referring to the calculations of previous studies [12], we calculated the F-statistic for each IV and excluded from further analyses those IVs with an F-statistic < 10. Finally, to avoid exposure-related SNPs acting on the outcome through confounders, SNPs that met the above criteria were further screened with the Phenoscanner V2.

### 2.4. MR Estimate

In our two-sample MR analysis, the primary method employed is the inverse-variance weighted (IVW) approach, which operates under the assumption that all IVs are valid, ensuring high efficacy. Additionally, we incorporated the MR Egger and Weighted Median methods as supplementary analyses. MR Egger assumes that all IVs are invalid, while Weighted Median assumes that only half of the IVs are valid. These supplementary methods provide a robustness check and offer insights into the potential impact of invalid instruments, enhancing the comprehensiveness of our analysis [13]. A *p* < 0.05 was used to determine the existence of an association between the exposure and outcome phenotype. Since serum uric acid levels are continuous data, the estimated effect value was assessed as β (95% confidence interval), whereas the odds ratio (95% confidence interval) was used for gout, as it is dichotomous data.

### 2.5. Sensitivity Analysis

**Figure 2:**
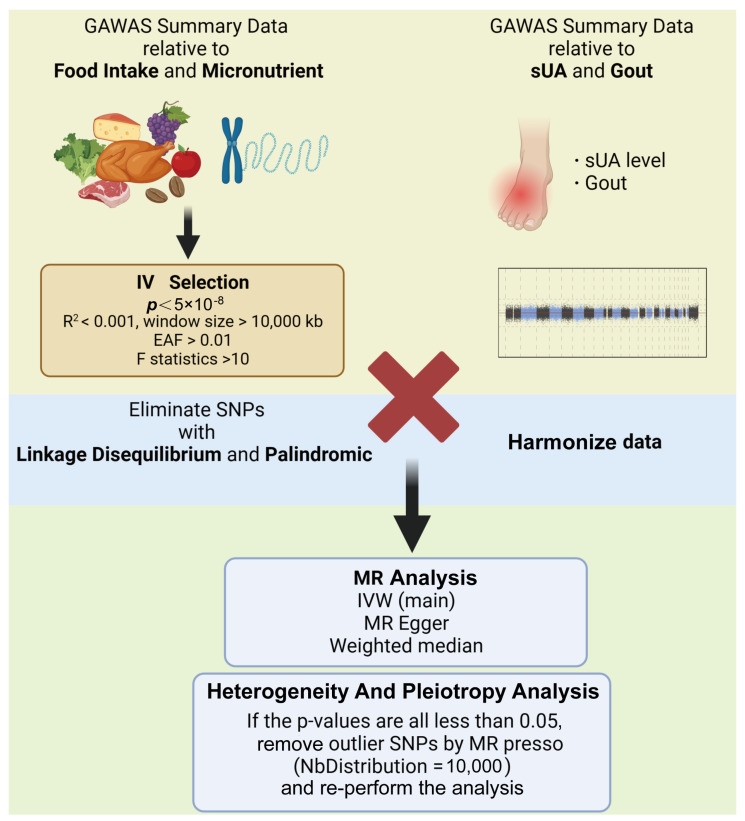
Analyzing processes (created with BioRender.com) (IVW, inverse-variance weighted; sUA, serum uric acid).

Cochran’s Q test was utilized to assess the heterogeneity of the analyzed results, while the Egger intercept test was applied for the pleiotropy analysis. In the MR analysis, due to the high impact of horizontal pleiotropy on the reliability of the results, MR presso with NbDistribution set to 10,000 was used to get rid of outlier SNPs for results with horizontal pleiotropy, and then a secondary analysis was performed. In addition, positive results obtained from two-sample MR analyses required further leave-one-out analyses to test the robustness of the results by getting rid of each SNP included in the analysis individually. All data analysis procedures were carried out using R software (version 4.3.1) and the TwoSample MR packages, ensuring comprehensive and robust analyses (Figure 2).

### 2.6. Estimation of Sample Overlap

Sample overlap between exposure and outcome data can bias the results of MR analyses; therefore, we tried to make them originate from different cohorts. However, there was a small sample overlap between the exposure data of macronutrients and the outcome data of gout in this study. To assess whether these sample overlaps would have interfered with the results of the analyses, estimates of bias and type I error incidence were performed with reference to the method described by Stephen Burgess [14].

## 3. Results

After rigorous screening, the analysis incorporated a range of IVs numbering between 4 and 88, all possessing an F-statistic greater than 10 (Supplementary Materials). An assessment of sample overlap showed that the maximum sample overlap between exposed macronutrient and the ending gout was 3.16%, with an expected resulting analytic bias < 0.001 and a type 1 error rate of 0.05, suggesting that a small amount of sample overlap would not significantly affect the results of the analysis.

The IVW approach was used as the primary method based on a *p* < 0.05 and for results without horizontal pleiotropy. We determined that non-oily fish intake, cheese intake, and sugar intake were associated with altered serum uric acid levels, whereas alcohol intake frequency, tea intake, coffee intake, poultry intake, and dried fruit intake were associated with the occurrence of gout, suggesting that they are risk/protective factors for gout.

### 3.1. Causality between Non-Oily Fish Intake and Serum Uric Acid Level

**Figure 3:**
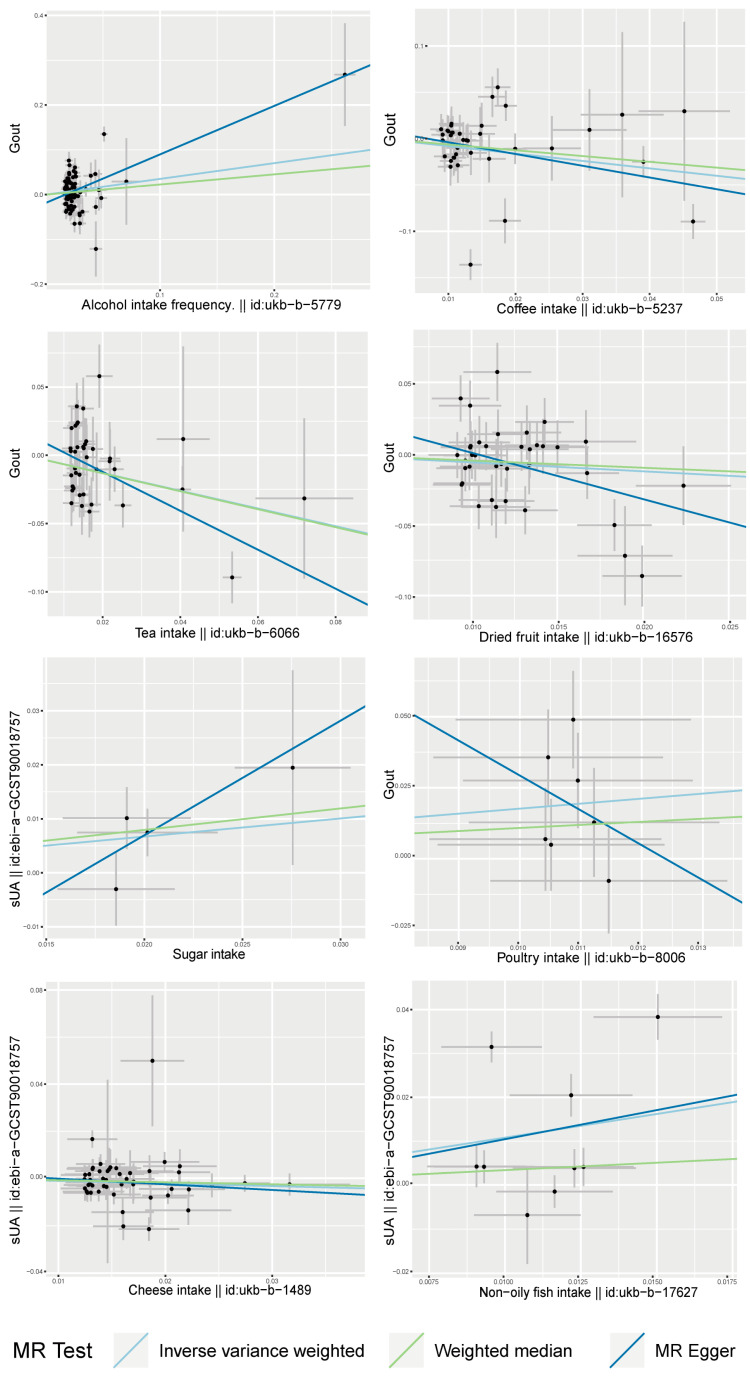
Scatter plots of MR analysis of dietary factors significantly associated with serum uric acid or gout (based on IVW, MR Egger, and Weighted Median) (sUA, serum uric acid).

**Figure 4:**
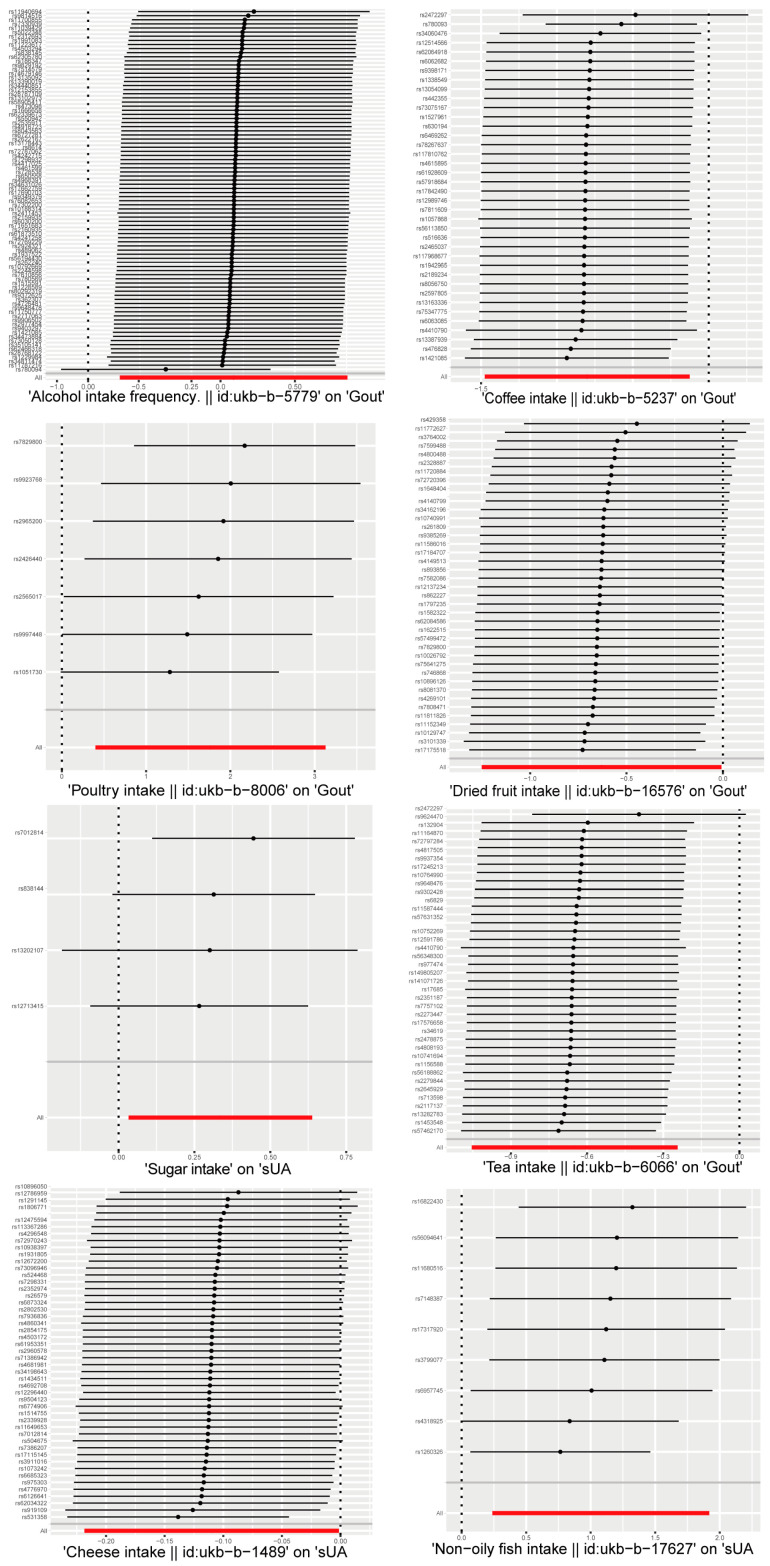
Leave-one-out sensitivity analysis of dietary factors significantly associated with serum uric acid or gout. (Each line indicates the estimated effect value and its confidence interval after getting rid of this SNP. The "ALL" in the bottom row indicates the combined estimated effect value and its confidence interval for all SNPs.)

After screening, a total of nine SNPs were included in the analysis. The analysis based on the IVW method revealed that non-oily fish intake was significantly associated with elevated serum uric acid levels (*p* = 0.012). The estimated effect value (β = 1.079) suggests that for every 1-unit increase in non-oily fish intake, there is a subsequent elevation of serum uric acid levels by 1.079 units. The two supplemental analysis methods are shown in Figure 3 to have consistent effect value directions with the IVW method. The leave-one-out analysis plot in Figure 4 suggests that the results of the analyses derived from these nine SNPs are robust.

### 3.2. Causality between Sugar Intake and Serum Uric Acid Level

Four SNPs were included in the causal inference of the association between sugar intake and serum uric acid levels. The main analysis method, IVW analysis (*p* = 0.030), as well as Weighted Median (*p* = 0.049) suggested a correlation between the two. The estimated effect value (β = 0.335) suggests that for every 1-unit increase in dietary sugar intake, there was a 0.335-unit increase in serum uric acid levels. However, due to the inclusion of only four SNPs, the robustness of the relationship inferred was low, as seen in the leave-one-out analysis (Figure 4).

### 3.3. Causality between Cheese Intake and Serum Uric Acid Level

A total of 48 SNPs were included in the causal inference of the relationship between cheese and serum uric acid levels. A correlation was found by the IVW method (*p* = 0.047). The estimated effect value (β = −0.110) suggested that cheese intake was a protective factor with reduced serum uric acid levels; an increase of 1 unit of cheese intake can downregulate serum uric acid levels by 0.11 units. A consistent direction of the effect value was also obtained by the two complementary analysis methods (Figure 3). The leave-one-out analysis plot in Figure 4 shows the overall robustness of the results of the analysis.

### 3.4. Causality between Alcohol Intake Frequency and Gout

A total of 88 SNPs were eligible for inclusion in the analyses based on the aforementioned screening criteria. The analysis by the IVW method found that the alcohol intake frequency was significantly associated with the attack of gout (*p* = 0.012). The estimated effect value (OR = 1.422) implies that the risk of the attack of gout was increased 1.422 times for every 1-unit increase in the alcohol intake frequency. The two supplemental analysis methods are shown in Figure 3 to have consistent directions of effect with the main analysis method. The leave-one-out analysis plot in Figure 4 suggests that the results of the analyses derived from these 88 instrumental variables are largely robust, with rs780094 making the largest contribution to the significant effect between the two.

### 3.5. Causality between Tea Intake and Gout

After screening, 39 SNPs were included to infer causality between tea intake and gout attacks. Tea intake was found to be a protective factor in suppressing gouty attacks by the IVW method (*p* = 0.002), MR Egger (*p* = 0.002), and Weighted Median (*p* = 0.016), and the estimated effect value direction was consistent among the three analyses (Figure 3). The OR = 0.522 suggests that an increase of 1 unit of tea intake reduces the risk of gout to about half of the former level. Moreover, this result was analyzed by the leave-one-out method and found to be overall robust, with rs2472297 contributing more to the significant relationship.

### 3.6. Causality between Dried Fruit Intake and Gout

A total of 39 SNPs were eligible for inclusion in the analyses. The IVW method analysis identified dried fruit intake as a protective factor for reducing gouty attacks (*p* = 0.047) with an estimated effect value of OR = 0.533. The two supplemental analysis methods are shown in Figure 3 to have consistent directions of effect with the main analysis method. And the leave-one-out analysis plot in Figure 4 suggests that the results of the analyses derived from these 39 instrumental variables are robust.

### 3.7. Causality between Coffee Intake and Gout

A total of 38 SNPs were included in the causal analysis of coffee intake and gout attack, and it was found that coffee intake was a protective factor for lowering the incidence of gout (P_IVW_ = 0.020); in addition, threshold correlations were found in the other two complementary analyses (*p*_MR Egger_ = 0.078, *p*_Weighted Median_ = 0.074), and the estimated effect value direction of the complementary analysis was consistent with the main analysis method (Figure 4). The estimated effect value OR = 0.449, which means that for every 1-unit increase in coffee intake, the risk of gout is reduced to approximately half of its original value. The leave-one-out method of analysis plot showed the overall robustness of the analysis, with rs2472297 contributing more to the significant relationship between the two.

### 3.8. Causality between Poultry Intake and Gout

Poultry intake was found to be associated with the risk of gouty attack (P_IVW_ = 0.011) by the causal inference of seven screened SNPs. The estimated effect value (OR = 1.764) suggested that poultry intake was a risk factor for gouty attacks, with a 1.764-fold increase in the risk of gouty attacks for every 1-unit increase in its intake. The leave-one-out analysis suggested the overall robustness of the analysis (Figure 4). However, when analyzed by the complementary method (MR Egger), inconsistent directions of the effect value were found (Figure 3).

## 4. Discussion

**Figure 5:**
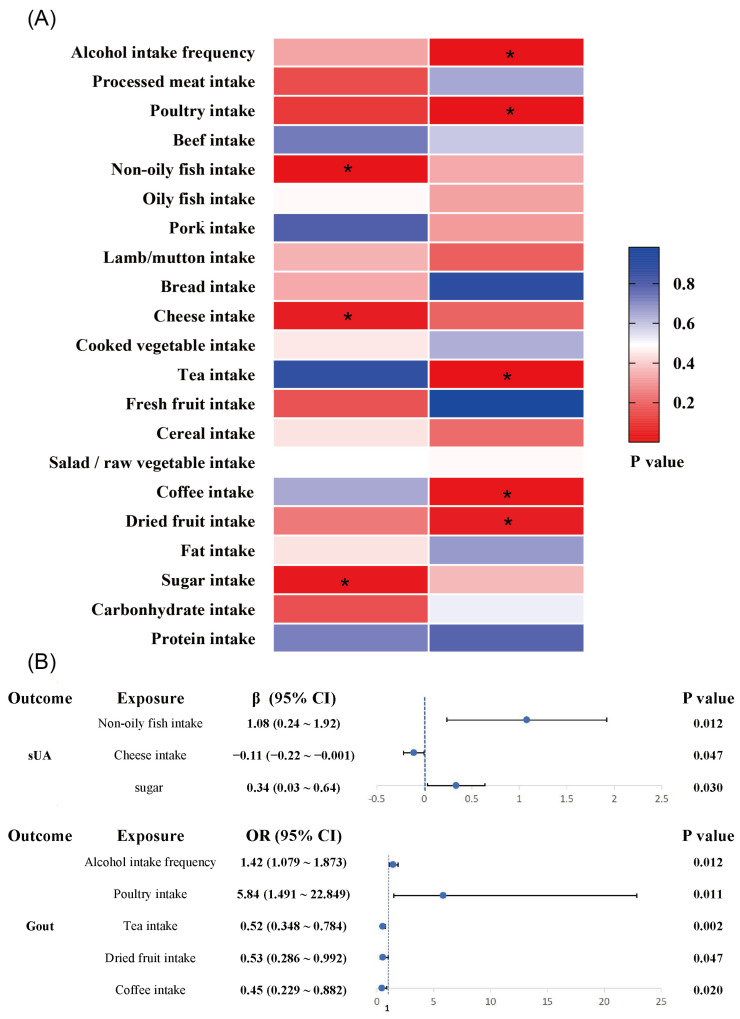
Risk/protective factor analysis of dietary intake based on IVW method: (**A**) heat map of the correlation between dietary factors and serum uric acid/gout (*, *p* < 0.05); (**B**) MR analysis of risk/protective factors associated with serum uric acid and gout. (sUA, serum uric acid; CI, confidence interval).

In this study, five common daily foods were identified to be associated with the risk of gout attacks by the genetic inference method of MR analysis (Figure 5): alcohol intake frequency (OR = 1.422, [95%CI: 1.079~1.873], *p* = 0.012) and poultry intake (OR = 5.836, [95%CI: 1.491~22.849], *p* = 0.011) were risk factors for the incidence of gout, whereas dried fruit intake (OR = 0.533, [95%CI: 0.286~0.992], *p* = 0.047), tea intake (OR = 0.523, [95%CI: 0.348~0.784], *p* = 0.002), and coffee intake (OR = 0.449, [95%CI: 0.229~0.882], *p* = 0.020) were protective factors in inhibiting the incidence of gout. The elevated serum uric acid value is the primary risk factor for gout, and this study found that non-oily fish intake, cheese intake, and sugar intake were associated with serum uric acid levels. Non-oily fish intake (β = 1.08, [95%CI: 0.24~1.92], *p* = 0.012) and sugar intake (β = 0.34, [95%CI: 0.03~0.64], *p* = 0.030) were risk factors for elevated serum uric acid levels, while cheese intake (β = −0.11, [95%CI: −0.22 ~ −0.001], *p* = 0.047) was associated with decreased serum uric acid levels.

At present, there is a notable absence of extensive randomized controlled trials examining the correlation between dietary intake and the occurrence of hyperuricemia or gout. Instead, the existing body of research predominantly relies on cross-sectional and retrospective survey methodologies. Aligned with our research, certain dietary patterns have demonstrated the potential to mitigate the risk of hyperuricemia and gout. Notably, coffee consumption has been associated with diminished serum uric acid levels and a reduced likelihood of gout, particularly among the male population [15,16]. Xanthine oxidase (XOD) is a key enzyme in the production of uric acid, and existing studies have found that the coupling products of caffeic acid and vinylcatechol identified from roasted coffee beans were found to have XOD inhibitory effects [17]. A cross-sectional study from China found that tea intake was negatively associated with the risk of hyperuricemia [18]. There are various active compounds in tea, such as polyphenols, alkaloids, pigments, etc. Existing studies have found that tea and its compounds can inhibit the activity of XOD and regulate the expression of the uric acid transporter proteins URAT1, GLUT9, and ABCG2, which can play a therapeutic role in the treatment of hyperuricemia [6]. A prospective study covering 4449 older adults found that fish and seafood consumption was positively associated with the incidence of hyperuricemia [19]. A diet rich in fiber, exemplified by increased fruit intake, has proven to be a protective factor against the development of gout [20]. The U.S. National Health and Nutrition Examination Survey, which included 41,230 participants, found that poultry intake was associated with a higher risk of hyperuricemia, while fruit and dairy intake were negatively associated with the risk of hyperuricemia [21]. Additionally, the consumption of dairy products, particularly cheese, has shown promise in reducing the risk of gout, as evidenced by a comprehensive retrospective study involving over 47,000 individuals [22]. Conversely, certain dietary practices have been identified as potential risk factors for these conditions. Notably, findings from a Chinese survey underscored that alcohol consumption contributes to an increased risk of gout [20]. Another survey conducted in China, encompassing over 3000 adolescents, revealed a positive correlation between poultry consumption and elevated serum uric acid levels [23]. However, our findings are also inconsistent with the conclusions reached in some extant clinical studies. A questionnaire from the rural areas of South Korea that included more than 9000 people failed to find an association between coffee intake and hyperuricemia [24]. Moreover, the Third National Health and Nutrition Examination Survey (1988–1994) in the United States found that tea intake was not beneficial to the reduction of serum uric acid levels [15]. However, the areas in which these two studies were conducted are not major coffee- or tea-consuming areas, and the results of these two clinical investigations should be accepted with caution. Differences in dietary habits across regions may act as confounders affecting the results of observational studies, and excluding the effects of confounders is the strength of the MR analysis.

As for studies on the effects of macronutrient intake (protein, fat, carbohydrates, and sugar) in food on gout and hyperuricemia, the available evidence suggests that sugar-sweetened beverages are associated with an increased risk of gout and hyperuricemia [25]. This corroborates the finding in our study that high sugar intake is a risk factor for elevated serum uric acid levels. In addition, both low-fat diets and low-carbohydrate diets have been found to reduce serum uric acid levels in patients with hyperuricemia [26]. However, another clinical study showed that high-carbohydrate diets had a risk of increasing serum uric acid values only when such foods possessed a high glycemic index [27]. High-carbohydrate diets were also not found to be a risk factor for such diseases in this research. Finally, although protein is used as a source of purines, clinical studies have found that a high-protein diet can help reduce serum uric acid levels in hypertensive patients [28]. A prospective study that included 47,150 participants with no history of gout similarly found that high protein intake did not increase the risk of gout [22].

Despite the plethora of clinical studies examining the impact of dietary factors on serum uric acid levels and gout, retrospective investigations are often encumbered by limitations in their ability to rigorously control for confounding variables. The inherent challenge lies in the complexity of daily dietary exposure, making it impractical to isolate the effects of individual food elements within the constraints of a randomized controlled trial. The multifaceted nature of dietary exposure makes it susceptible to confounding factors, necessitating alternative analytical approaches. In response to these challenges, the MR analysis becomes crucial for assessing the potential risk or protective role of dietary factors in patients with gout and hyperuricemia. The MR analysis, by leveraging genetic variation as instrumental variables, offers a more robust methodology for estimating causal relationships between dietary exposures and disease outcomes.

It is of concern that although some dietary risk factors strongly associated with gout were identified by the MR analysis, a more in-depth interpretation of the results is needed. Dried fruit intake was analyzed as a protective factor for gout. Existing studies have found that polyphenols, vitamin C, dietary fiber and minerals, which are abundant in fruits, can exert a uric-acid-lowering effect, playing a role in lowering uric acid levels by inhibiting XOD activity, promoting uric acid excretion, and inhibiting uric acid reabsorption [29]. However, some fruits are rich in fructose, and high fructose intake is considered to be a risk factor for elevating serum uric acid levels and inducing hyperuricemia [30]. Therefore, the interpretation of this result should be limited to those fruits that are low in fructose and contain more uric-acid-lowering actives, such as peaches, apples, lemons, oranges, apples, etc. [29]. The purine content of fish is more than 200 mg/100 g, which is traditionally regarded as a purine-rich food [31]. Using an MR analysis, we found that non-oily fish intake was a risk factor for elevated serum uric acid levels, and it is of interest to note that there was not such an association for oily fish intake. Fish oil may be a key ingredient in the prevention of gouty attacks, as it is rich in the omega-3 fatty acid, which has been widely reported to have anti-inflammatory activities [32]. Clinical research has found that fish oil supplementation contributes to lower serum uric acid levels and reduces the risk of gout [33,34]. In addition, this study found that poultry intake was a risk factor for gout by using the main method, IVW analysis. However, the results analyzed by the MR Egger method showed a different direction of the effect value; therefore, the existence of a causal relationship cannot be concluded from the perspective of genomic association, based on the results of the current analyses [35].

Finally, it is imperative to acknowledge certain limitations in our study. Since the MR analysis is a secondary analysis based on the results of a large-scale GWAS study, this has to be limited by its study design. Notably, our research instrument could not differentiate between genders, despite the prevailing understanding that gout exhibits a higher prevalence in males. Secondly, serum uric acid values are in a dynamic state of change, and multiple time-points of measurement are needed to better assess the true level of uric acid [36]. Furthermore, the majority of our GWAS data originated from European populations, indicating a limitation in the absence of comprehensive population-wide mass sequencing data. These constraints underscore the necessity for future studies to address such limitations and enhance the generalizability and applicability of findings across diverse populations.

## 5. Conclusions

In conclusion, our study has successfully identified several dietary factors associated with serum uric acid levels and gouty attacks. Notably, cheese intake was associated with lower serum uric acid levels, and tea, coffee, and dried fruit intake showed a preventive effect on the risk of gouty attacks. In contrast, non-oily fish and sugar intake were risk factors for elevated serum uric acid levels, and alcohol intake frequency was a risk factor for gout predisposition. These findings are consistent with some of the existing observational studies. By inferring the role of daily dietary factors in gout by means of Mendelian randomization analyses, the present study can provide evidential support for existing clinical studies on the effects of missing strictly conditionally controlled dietary factors on gout onset and provide valuable insights for the development of nutritional strategies for patients with hyperuricemia and gout.

## Data Availability

The data regarding food intake and serum uric acid level related phenotypes were obtained from the IEU open GWAS project database (https://gwas.mrcieu.ac.uk/) (accessed on 15 January 2024). Information on SNPs related to macronutrients was obtained from the GAWS literature (doi:10.1038/s41380-020-0697-5.). Gout phenotypes were extracted from the FinnGen database (https://www.finngen.fi/fi) (accessed on 15 January 2024).

## Supplementary Materials

The following supporting information can be downloaded at https://www.mdpi.com/article/10.3390/foods13081269/s1.
